# What is the Role of Coronary Physiology in the Management of Patients with Chronic Coronary Syndromes?

**DOI:** 10.31083/j.rcm2304145

**Published:** 2022-04-13

**Authors:** Alec Saunders, Nick Curzen

**Affiliations:** ^1^Faculty of Medicine, University of Southampton, SO17 1BJ Southampton, UK; ^2^Cardiothoracic Care Group, University Hospital Southampton NHS Trust, SO16 6YD Southampton, UK

**Keywords:** coronary artery disease, coronary physiology, myocardial ischemia, coronary angiography

## Abstract

The use of coronary physiology in patients with chronic coronary syndromes is highly variable, and the evidence base complex. Tests of coronary physiology have traditionally been invasive (e.g., fractional flow reserve), but novel non-invasive methods are now available which provide additional anatomical information (e.g., computed tomography-based fractional flow reserve and angiogram-derived physiology). This review summarises the evidence for and against the relative value of these tests for patients being investigated for chest pain that may represent chronic coronary syndromes, and for those triaged to percutaneous coronary intervention.

## 1. Introduction

### Chronic Coronary Syndrome: Background Challenges Regarding Optimal Investigation and Management Strategies

Coronary artery disease (CAD) is associated with the accumulation of atherosclerotic plaque in epicardial arteries and it presents as an acute or chronic coronary syndrome (CCS) [1]. CAD was the most common cause of death globally in 2019 and it affects over 2 million people in the United Kingdom alone [2, 3]. The accurate and efficient investigation and management of patients with CCS is therefore of considerable importance.

Investigation strategies for CCS rely on evaluation of (i) an imaging test (burden of atheroma), (ii) coronary physiology (burden of ischaemia), or (iii) both. Anatomical evaluation for the detection of atheroma has traditionally been performed using invasive coronary angiography (ICA), but advances in the diagnostic accuracy of computed tomography coronary angiography (CTCA) means that this test is able to identify atheromatous lesions with a similar level of accuracy in most cases [4]. However its application is limited in some patient groups, for example those with tachyarrhythmias or high body mass index. Invasive or non-invasive assessment of plaque vulnerability can additionally be used to provide further prognostic information [5, 6, 7, 8].

Physiological evaluation for the detection of ischaemia, or surrogates for ischaemia, can be achieved using a variety of tests. Non-invasive investigations include stress cardiac magnetic resonance, stress echocardiography, nuclear myocardial perfusion scans and now, only rarely, exercise tolerance tests [1]. Invasive tests for surrogates of ischaemia have traditionally relied on the intracoronary pressure wire, either using fractional flow reserve (FFR) or non-hyperaemic indices such as instantaneous wave-free ratio (iFR) [9]. More recently, complex computer models of fluid dynamics and 3D reconstruction have facilitated tools that provide surrogates for ischaemia either non-invasively, from the dataset created by a CTCA in the form of ${\mathrm{FFR}}_{\text{CT}}$, or from the invasive angiogram itself.

Given the diverse nature of the currently available tests with which to investigate patients presenting with new onset chest pain, and the rapidly increasing body of evidence, which has changed substantially recently, this review will address the relative pros and cons of the various approaches: invasive or non-invasive; anatomical or physiological?

Current clinical guidelines are discrepant in their recommendations, particularly in relation to their preference for anatomical or physiological testing of patients with CCS. The National Institute for Health and Care Excellence (NICE) CG95 guidelines (Chest Pain of Recent Onset) favour the use of CTCA for the investigation of the vast majority of patients with stable chest pain, except those with confirmed CAD [10]. By contrast, the European Society of Cardiology guidelines (2019 ESC Guidelines for the diagnosis and management of chronic coronary syndromes) encourage estimation of the pre-test probability of CAD based on the patient’s clinical presentation and risk factors [1]. Specifically, they recommend that those with a low clinical likelihood of obstructive CAD should be investigated with a CTCA, and those at greater risk should receive testing for ischaemia with either a functional non-invasive test or ICA with FFR/iFR. Invasive physiological assessment is particularly favoured for patients undergoing ICA with coronary stenoses of 50–90% or multivessel disease, where a mismatch between the angiographic and functional severity of a lesion is common [1].

Management plans for patients with CCS are derived from the results of these investigations. This decision-making process is complex, and must consider the merits of optimal medical therapy (OMT) versus revascularisation, percutaneous coronary intervention (PCI) versus coronary artery bypass grafting (CABG) and which vessels require intervention. There is considerable, and recently discrepant, evidence about whether these decisions and the subsequent clinical outcomes are improved by using surrogates of ischaemia on top of angiographic appearance to inform them.

The initial dilemma facing clinicians tasked with assessing and managing patients with stable chest pain is whether to use a test of atheroma burden, ischaemia burden, or both. The ideal decision pathway is dominated by the need to provide optimal patient care but also is required, in most local health economies, to be demonstrably cost effective.

## 2. The Case for Anatomical Tests

There is a plausible and logical case to be made that starting with a test of coronary anatomy that incorporates an overall assessment of atheroma burden and locality is dominant. To begin, such an approach establishes two fundamentally important facts: (a) is there an aberrant course for a coronary artery (usually between the aorta and pulmonary artery) that could explain chest pain and identify risk? And (b) are the coronaries clear of any atheroma? In patients who have no atheroma, classical angina can be excluded, and the implications regarding the lack of requirement for optimal medical therapy (OMT) and revascularisation are profound. The caveat to this is the expanding awareness of the prevalence of microvascular angina, which will not be addressed further in this review given limitations of space.

In patients who do prove to have a significant burden of atheroma, this provides a clear cut indication for the application of OMT. In this context, OMT comprises two components. Firstly, disease-modifying therapy including aspirin and a statin, the evidence for which are reviewed in reference [11]. Furthermore, given the results of the HOPE and EUROPA trials, there is an indication for ACE Inhibitors in most patients with coronary atheroma, regardless of left ventricular function [12, 13]. Secondly, anti-anginal drugs that are normally headed by beta blockers. The importance of OMT in the management and outcome of patients with CCS is well established by a variety of evidence sources. Apart from the data summarized in reference [11], the ability of this approach to yield clinical advantage is well established in the SCOT HEART trial [14]. In SCOT HEART, 4146 patients with stable chest pain were randomised to either standard care alone or CTCA as their first line test. After 5 years, the combined rate of death from CAD or non-fatal myocardial infarction (MI) was significantly lower in the CTCA group (hazard ratio 0.59; 95% CI 0.41–0.84) [15]. This was achieved despite similar overall rates of revascularisation, suggesting that the improved outcomes were due to better detection of CAD and the subsequent application of disease-modifying medical therapy [16]. It should be noted that the superior clinical outcome observed in the 5 year follow up was driven by non-fatal MI, rather than mortality. This may prove to be important, given the recent conflicting data about whether spontaneous myocardial infarction during follow up of patients with CAD is associated at some point with mortality.

On top of this evidence that favours a primarily anatomical approach linked with OMT, the results of the ISCHEMIA trial can be interpreted as supporting the concept that we could perhaps miss out ischaemia testing for most patients with stable angina in favour of this simpler algorithm [17]. This concept admittedly requires lateral thought and extrapolation, and we must also consider the limitations of the study including slow recruitment and lower than expected event rates. In ISCHEMIA, patients with stable angina were actually only meant to be included in the trial if they had at least moderate ischaemia burden at baseline. In fact, just over 10% had mild ischaemia or none at all. However, the trial reported that early angiography and revascularisation had no overall outcome advantage (using the complex primary composite endpoint of cardiovascular death, MI, hospitalisation for unstable angina or heart failure, and resuscitated out of hospital arrest) above and beyond application of OMT alone. Perhaps this population of patients with stable chest pain could have merely been triaged by CTCA alone as having important CAD and then been put on OMT without needing any other tests?

The power of OMT in its own right in the SCOT HEART and ISCHEMIA populations, and the outcome comparison to revascularisation in ISCHEMIA, makes a straightforward case for leading with detection of atheroma and application of OMT in all such patients as the default initial strategy. Later on in this review, we discuss a comparison of the prognostic value of atheroma burden versus ischaemia burden, but first we must review the evidence that ischaemia burden is of clinical value.

## 3. The Case for Tests of Ischaemia

### 3.1 Circumstantial Evidence that Ischaemic Burden Is Prognostically Important

There is a persuasive body of evidence that collectively indicates that the burden of ischaemia is indeed associated with prognosis, although nearly all these data present composites of death plus MI, and analysis shows that significant outcomes are almost always driven by the MI component, rather than by mortality. For example, in patients with stable chest pain and coronary artery lesions who received myocardial perfusion imaging using stress single photon emission computed tomography (SPECT) sestamibi, annual rates of death or nonfatal MI were 12 times higher in those with ischaemia than in those with normal images (7.4% vs. 0.6%) [18]. The relationship between ischaemia burden and prognosis was also demonstrated in the COURAGE nuclear substudy, which recruited patients with significant stable CAD and evidence of ischaemia [19]. They underwent myocardial perfusion SPECT imaging before and at 6 to 18 months after treatment with either OMT and PCI, or OMT alone. An almost linear relationship was present between the risk of death or MI and the extent and severity of residual ischemia at the second scan. This ranged from 0% for patients with no ischemia, to 39.3% for patients with 10% or greater residual ischaemia of the myocardial mass at follow up.

Observational data also show that the extent of myocardial ischaemia is associated with different survival rates for patients treated with OMT or revascularisation. For example, a study of over 10,000 consecutive patients undergoing exercise or adenosine stress myocardial perfusion SPECT imaging showed a positive association between the amount of inducible ischaemia and cardiac death rates for patients treated with OMT. This relationship was attenuated by revascularisation. Consequently, OMT was preferrable for patients with no inducible ischaemia (cardiac death rate 0.7% vs. 6.3%, statistically non-significant *p* value) and revascularisation was preferable for patients with greater than 20% myocardial ischaemia (cardiac death rate 2% vs. 6.7%, *p *< 0.0001) [20].

### 3.2 Evidence that Detection of Vessel-Specific Ischaemia Using the Intracoronary Pressure Wire Is Prognostically Important

The pressure wire provides an extremely well validated surrogate for downstream myocardial ischaemia based upon the measured pressure drop across lesion(s) in a vessel, either in the form of FFR or iFR. The basic point of added value to obtaining information regarding vessel-specific, and, more recently, lesion-specific ischaemia, is that, outside the context of acute ST elevation MI, implantation of coronary stents has value *only* in lesions responsible for downstream ischaemia. This has been shown in a series of high quality randomised trials including DEFER, FAME and FAME2 [21, 22, 23].

The DEFER trial (Fractional Flow Reserve to Determine the Appropriateness of Angioplasty in Moderate Coronary Stenosis) was the first randomised controlled trial exploring the use of FFR to direct PCI [21]. It recruited 325 patients referred for elective PCI with angiographically “significant” stenosis (>50% diameter stenosis) and no documented ischemia. FFR was measured prior to intervention. Those with haemodynamically insignificant lesions (defined as FFR >0.75) were randomised to either deferral or performance of PCI. Those with haemodynamically significant lesions (FFR <0.75) had PCI performed as planned. Freedom from angina was significantly higher following PCI of functionally significant lesions compared to functionally normal lesions. In patients with normal FFR, performance of PCI did not improve the rate of adverse cardiac events or freedom from angina compared to deferral of PCI. 15-year follow up data showed equal mortality rates between the three groups and a significantly higher rate of MI in patients with normal FFR in the perform group compared to the defer group [24]. The simple implication of DEFER is that lesions, however tight, do not benefit from PCI unless they are associated with downstream ischaemia, and do better if treated with OMT alone. More recent evidence suggests that deferral of revascularisation of non-ischaemic lesions can safely be performed using either iFR or FFR, as demonstrated in subsequent randomised trials such as DEFINE-FLAIR (Functional Lesion Assessment of Intermediate Stenosis to Guide Revascularisation) and iFR-SWEDEHEART (Instantaneous Wave-Free Ratio Versus Fractional Flow Reserve in Patients with Stable Angina Pectoris or Acute Coronary Syndrome) [25].

The FAME (Fractional Flow Reserve versus Angiography for Guiding Percutaneous Coronary Intervention) and FAME2 (Fractional Flow Reserve–Guided PCI versus Medical Therapy in Stable Coronary Disease) randomised trials also provide clear evidence of clinical benefit for the use of coronary physiology using FFR in populations who have been triaged to PCI based upon their angiographic appearances [22, 23]. FAME recruited over 1000 patients with multivessel CAD due to undergo PCI of lesions based on angiographic appearance. Patients were randomised to angiographically-guided PCI of all indicated lesions, or FFR-guided PCI of only those lesions with FFR ≤0.8. The primary composite endpoint of death, MI, and repeat revascularization was significantly lower in the FFR-guided group. Differences in rates of MI (8.7% vs. 5.7%, relative risk 0.66, *p* = 0.07) and repeat vascularisation (9.5% vs. 6.5%, *p* = 0.08) were more pronounced than those for mortality (3.0% vs. 1.8%, *p* = 0.19). These improved outcomes in the FFR-guided group were achieved despite (i) fewer stents being placed per patient (2.7 ± 1.2 vs. 1.9 ± 1.3, *p *< 0.001) and (ii) lower procedure-related costs. This occurred because over one in three of the (angiographically “significant”) lesions in the FFR group were haemodynamically normal and were hence left unstented.

The FAME2 study explored whether patients with functionally significant stenosis (FFR ≤0.80), suitable for PCI, would derive greater benefit from PCI with OMT or OMT alone. The study was halted prematurely after enrolment of 1220 patients, as the PCI group had significantly lower rates of the composite primary endpoint: death, MI, or urgent revascularization (4.3% vs. 12.7%, hazard ratio with PCI 0.32; 95% CI 0.19–0.53). The difference in this endpoint was chiefly driven by lower rates of urgent revascularisation (1.6% vs. 11.1%; hazard ratio 0.13; 95% CI 0.06–0.30).

For patients undergoing CABG, FFR-guidance does not appear to be quite so valuable. FFR-guided CABG results in fewer anastomoses per patient than for angiographically-guided procedures, but it does not carry any benefit in terms of major adverse cardiovascular event risk [26]. The recently published FAME3 study (Fractional Flow Reserve–Guided PCI as Compared with Coronary Bypass Surgery) recruited patients with angiographically identified three vessel disease. In this population, FFR-guided PCI was not shown to be non-inferior to CABG in terms of a composite of death, MI, stroke or repeat revascularisation at 1 year [27].

These trials have established some important principles in terms of the value of pressure wire measurement in patients who have already been triaged, on the basis of a diagnostic angiogram, to PCI. Firstly, stenting lesions that are non-ischaemic is associated with a worse outcome than treating them medically (DEFER), and that deferral of lesions that are non-ischaemic according to FFR or iFR is associated with a very good medium term outcome with low ischaemic event rates [21, 24, 25]. Secondly, that there is a lower event rate, driven by urgent revascularisation, in patients with pressure wire positive lesions if they are stented compared with if they are treated with OMT alone (FAME2) [23]. Finally, that pressure wire-directed multivessel PCI is associated with a significantly better clinical outcome (composite of death, MI and repeat revascularisation) than angiography-directed PCI, despite fewer stents being used in fewer vessels at lower overall cost in the former group (FAME) [22]. So, the ability of FFR (and iFR) to optimise PCI planning is extremely well established.

**Fig. 1.**
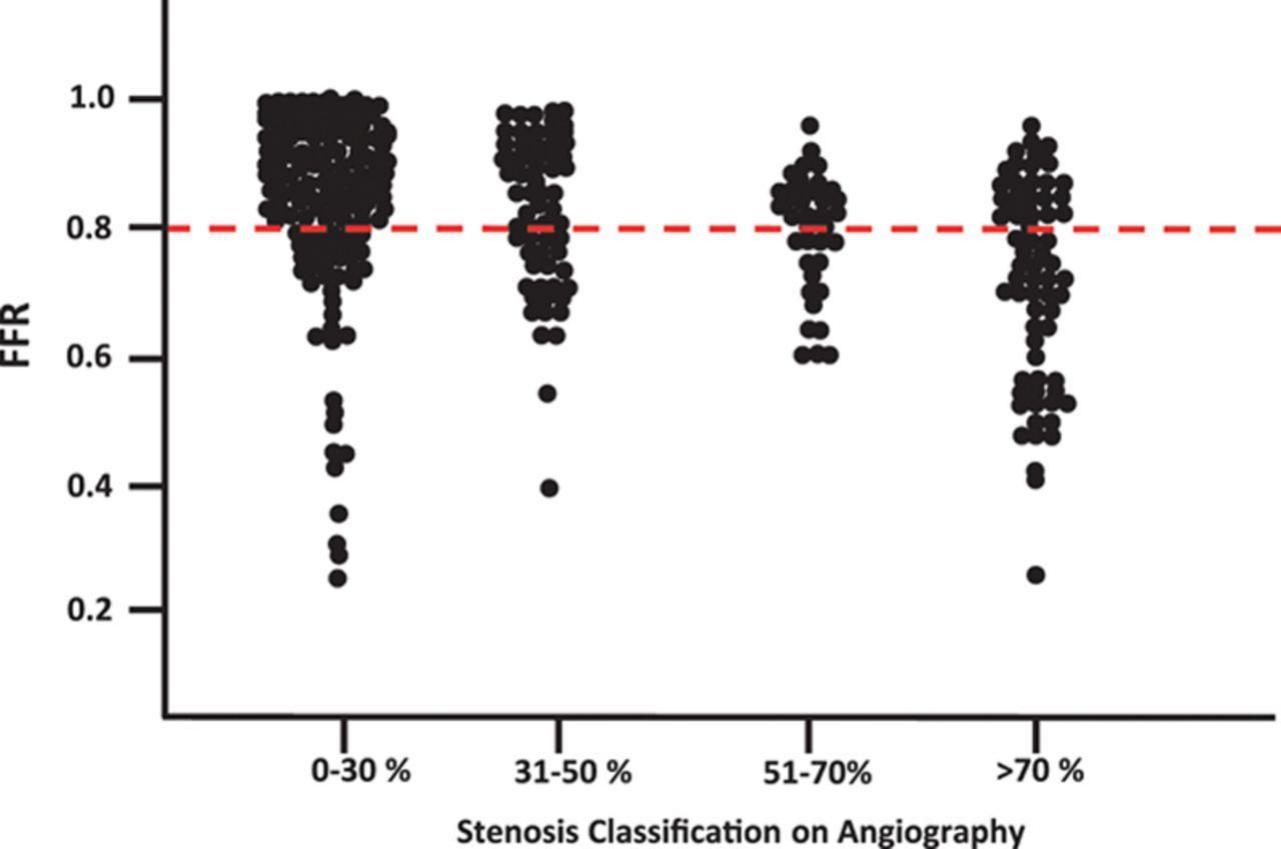
**Concordance between the angiographic severity of stenosis and physiological impairment assessed by fractional flow reserve**. Reproduced from [28].

By what mechanism does the pressure wire affect our assessment and management of our patients? The correlation between the angiographic appearance of a lesion (i.e., its “significance”) and whether or not it is capable of causing downstream ischaemia is not close, so that there is a discrepancy in about 30% of lesions (Fig. 1, Ref. [28]). This observation has been made in a variety of patient populations, and the degree of discordance is remarkably consistent. Not surprisingly, this discordance has profound implications for the subsequent management of the vessels, and therefore patients. For example, in RIPCORD, which included 200 patients undergoing a diagnostic angiogram for stable chest pain, there was a change in management (between OMT alone, PCI, CABG or more information required) in 26% of patients when FFR of all the major epicardial coronary arteries was available when compared to the plan based upon the angiographic appearances alone [28]. This observation is consistent with a variety of other non-randomised studies, which demonstrate that the availability of some pressure wire data changes the management of the population in between a quarter and a half of the patients because of this effect [29].

### 3.3 The Value of ${\mathrm{FFR}}_{\text{CT}}$: Having Non-invasive Anatomy and Assessment of Ischaemia in the Same Test

${\mathrm{FFR}}_{\text{CT}}$ is a well validated method for modelling FFR in all major epicardial coronary vessels using the dataset from CTCA together with other clinical parameters [30]. The test therefore provides a comprehensive assessment of the presence, severity and distribution of atheroma as well as vessel-specific ischaemia.

In the non-randomised PLATFORM study, 584 patients with new onset chest pain were enrolled into two consecutive cohorts [31]. Patients in the first cohort were assigned to receive usual testing. Those in the second cohort underwent CTCA instead of planned non-invasive or invasive testing, followed by ${\mathrm{FFR}}_{\text{CT}}$ if the CTCA showed 30% or greater stenosis or if the patient was referred to ICA. In the planned invasive cohort, 73% of patients in the usual care arm had no obstructive CAD on ICA compared to 12% in the ${\mathrm{CTCA/FFR}}_{\text{CT}}$ arm. After receiving ${\mathrm{CTCA/FFR}}_{\text{CT}}$ results, ICA was cancelled in 61% of cases in this arm. This was achieved without negatively impacting clinical outcomes. Specifically, no difference was present in major adverse cardiac event rate or quality of life between patients in either arm of the planned invasive cohort after 1 year of follow up [32]. Further, a prespecified analysis of the study demonstrated that ${\mathrm{FFR}}_{\text{CT}}$ was cost dominant in patients who would have undergone invasive coronary angiography [33].

The power of ${\mathrm{FFR}}_{\text{CT}}$ to direct management was further demonstrated in the ADVANCE registry, which included 5083 patients with clinically suspected CAD, who had atherosclerosis identified by the presence of >30% stenosis on CTCA [34]. The availability of ${\mathrm{FFR}}_{\text{CT}}$ results changed management plans in 66.9% of the patients. One of the most impactful observations was the reassuringly low clinical event rates of patients with coronary disease that was ${\mathrm{FFR}}_{\text{CT}}$ negative (43 major adverse cardiac events in patients with ${\mathrm{FFR}}_{\text{CT}}$≤0.80 vs. 12 in those with ${\mathrm{FFR}}_{\text{CT}}$>0.80), thus mirroring the invasive pressure wire data [35].

Based upon the positive observational data accrued about the value of ${\mathrm{FFR}}_{\text{CT}}$ in clinical practice, as well as economic modelling suggesting large cost savings, a NICE Technology Appraisal recommended the use of the test in front line clinical practice in the UK, and the cost was subsidised by NHS England [36]. As a consequence, there was widespread uptake of ${\mathrm{FFR}}_{\text{CT}}$ involving the majority of Trusts in the UK.

The main advantage that ${\mathrm{FFR}}_{\text{CT}}$ offers in routine practice is a rapid non-invasive assessment of atheroma and ischaemia burden. This combination facilitates decisions about the application of OMT, and planning of potential revascularisation strategies in those patients with ongoing angina. However, until recently there has been no randomised trial data available.

### 3.4 Shouldn’t an Assessment that Combines Anatomy and Physiology at the Diagnostic Stage Lead to Better Clinical Outcome and Lower Cost?

Given: (a) the association between overall ischaemic burden and outcome; (b) substantially improved outcomes for PCI directed by pressure wire with angiography when compared to angiographic assessment alone; (c) the ability of ${\mathrm{FFR}}_{\text{CT}}$ to facilitate assessment and management of patients with good clinical outcomes despite substantial reductions in the need for invasive angiography, there is a plausible and logical hypothesis that routine assessment of both anatomy and physiology of all epicardial coronary arteries would be associated with a better outcome at the diagnostic stage than assessment by angiography (invasive or CTCA) alone. Furthermore, given the economic analysis results from FAME (invasive) and PLATFORM (non-invasive), it would also be reasonable to speculate that such a strategy may prove cost dominant. This concept has now been tested in two randomised trials, one using invasive angiography and pressure wire assessment (RIPCORD2) and the other using ${\mathrm{FFR}}_{\text{CT}}$ (FORECAST) [37, 38].

The RIPCORD2 trial (Routine Pressure Wire Assessment Versus Conventional Angiography in the Management of Patients with Coronary Artery Disease) recruited 1100 patients undergoing ICA for the investigation of stable angina or non-ST elevation MI [37]. The key angiographic inclusion criterion was that participants were required to have at least one stenosis of 30% or greater in a coronary vessel of a calibre suitable for revascularisation. Patients were randomised to assessment and management based upon (a) angiographic appearances alone (ANGIO alone) or (b) angiographic appearance plus systematic FFR measurement in all epicardial vessels of sufficient size to be amenable to revascularization (ANGIO + FFR). Patients randomised to ANGIO + FFR had a median of 4 vessels investigated with FFR (interquartile range 3–5). As in the original study, this approach led to longer cases with greater contrast use, and a pressure-wire related complication rate of 1.8%. The routine use of FFR did not result in a significant difference in the co-primary endpoints of (i) total hospital costs and (ii) quality of life and angina status at 1 year. The rates of all-cause mortality, non-fatal stroke, non-fatal MI and unplanned revascularisation, the principal prespecified secondary endpoint, were also similar in both groups. The experimental strategy resulted in fewer patients requiring additional tests (1.8% vs. 14.7%, *p *< 0.00001), but it did not result in differences between groups in the proportion allocated to OMT, PCI or CABG. The RIPCORD2 result is consistent with both FUTURE and FLOWER MI in showing no benefit in systematic FFR-directed assessment and management of patients above and beyond their angiographic assessment *at the stage of diagnostic angiography* [39, 40]. This view is also supported by meta-analysis showing that in patients with STEMI and multi-vessel CAD, complete revascularisation guided by angiography but not FFR is associated with lower rates of recurrent MI [41].

The FORECAST trial (Fractional Flow Reserve Derived from Computed Tomography Coronary Angiography in the Assessment and Management of Stable Chest Pain) randomised 1400 patients with stable chest pain to either (a) initial testing with CTCA and selective ${\mathrm{FFR}}_{\text{CT}}$ for those with a stenosis of 40% or greater, or (b) standard clinical care based on NICE guidelines [38]. There was no significant difference in the primary endpoint of mean total cardiac costs at 9 months. Nor were there differences between the groups in clinical outcomes including quality of life, angina status and major adverse cardiac and cerebrovascular events or in the rate of revascularisation. However, ICA rates were 22% lower in the ${\mathrm{FFR}}_{\text{CT}}$ arm, and the proportion of invasive angiograms showing no obstructive epicardial lesion was 52% lower. Certainly, this study did not show that use of ${\mathrm{FFR}}_{\text{CT}}$ was associated with the considerable cost savings predicted by the NICE Technology Appraisal [36].

## 4. So, Which Is Dominant in CCS Patients: Atheroma or Ischaemia?

Clearly, this question is flawed, because assessment of anatomy and physiology are not mutually exclusive, and, in fact, in many cases can be considered complementary. However, recent data yields a picture that suggests that assessment of atheroma burden is dominant for the diagnostic assessment of patients with suspected CCS. The data presented above are consistent with this notion. SCOT HEART speaks of the power of OMT, without specific requirement for ischaemia testing. ISCHEMIA demonstrates that the optimal treatment for CCS patients is OMT unless they have breakthrough angina (and assuming that left main coronary stenosis has been excluded), and this obviously raises the logical question: why bother with the ischaemia testing in the first place for such patients? Entirely consistent with this, both RIPCORD2 and FORECAST demonstrate no advantage to routine assessment of ischaemia in patients requiring a diagnostic test for suspected CCS.

In addition to this convincing body of evidence, *post hoc* analyses of both PROMISE and ISCHEMIA provide some insight into why atheroma burden seems more important. The PROMISE trial randomised over 9000 patients with stable chest pain to CTCA or routine clinical assessment. In a follow up paper, the authors looked at the association between incremental burden of ischaemia and atheroma and the composite primary endpoint (death, MI or hospitalisation for unstable angina) [42]. Ischaemia was assessed using exercise electrocardiography, nuclear stress or stress echocardiography. When the test findings were stratified as mildly, moderately, or severely abnormal, hazard ratios (compared to a normal test result) increased proportionally for CTCA (2.94, 7.67, 10.13; all *p *< 0.001) but not for equivalent ischaemia categories (0.94 [*p* = 0.87], 2.65 [*p* = 0.001], 3.88 [*p *< 0.001]). The authors concluded that CTCA provided superior prognostic information than ischaemia testing in patients with stable chest pain. More recently, the ISCHEMIA investigators have published a similar analysis [43]. They presented 4 year outcomes according to strata of increasing atheroma and ischaemia burden, and found that there was a significant “dose response” for increasing atheroma burden and both mortality and MI. By contrast, neither the moderate nor severe ischaemic strata were associated with increased mortality, although MI was associated with the most severe ischaemia. These data are entirely consistent: in both trial populations, *total atheroma burden was a better predictor of adverse clinical events than ischaemia burden*. Such observations provide insight into the reason for the results of SCOT HEART, RIPCORD2 and FORECAST.

## 5. Conclusions and Suggestions for Clinical Practice

**Fig. 2.**
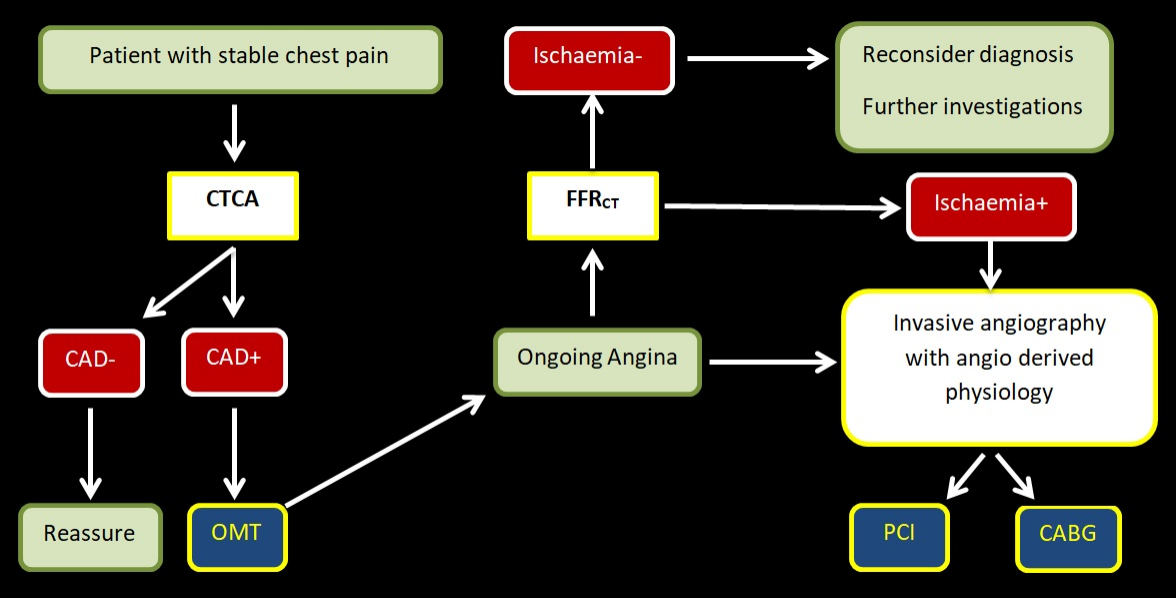
**Proposed pathway for the investigation and management of patients with stable chest pain**. Reproduced from [44].

The results of both RIPCORD2 and FORECAST do not support a routine assessment of ischaemia at the stage of the diagnostic angiogram. Further, whilst FORECAST yielded a significant reduction in the need for invasive coronary angiography, which is a valuable result both for patients and for hospital efficiency, neither trial demonstrated significant cost savings. The results are consistent with the notion that the initial assessment of patients with stable chest pain can be based around atheroma burden by CTCA, and that the initial management of patients found to have atheroma should then be OMT. This algorithm is consistent with the findings of ISCHEMIA, and has the consequence that only those patients with ongoing angina after full OMT is deployed need to be considered for revascularisation (Fig. 2, Ref. [44]). It is important to note, however, that if the chosen mode of revascularisation is PCI, then FFR or iFR guidance of this procedure would, indeed, be associated with a better, and cheaper, outcome.

In the future, tests of coronary physiology will become simpler and quicker to obtain. For non-invasive assessment, the computation of ${\mathrm{FFR}}_{\text{CT}}$ can now be performed in under 30 minutes using simplified modelling, and machine learning techniques can be applied to improve its diagnostic accuracy [45, 46]. For patients requiring invasive angiography, novel models provide angiogram-derived physiology. Measures such as quantitative flow ratio show good agreement with FFR values, but do not require the use of pressure wires or induction of hyperaemia [47]. A variety of other similar measures are available and some have already reached the market [48]. The true value of these technological advancements will be determined by finding the patients and clinical situations where they are best deployed. For the present, an initial CTCA followed by deployment of OMT seems to be dominant in CCS patients. The value of knowing vessel-specific ischaemia is, however, still extremely valuable in patients who have been committed to PCI.
